# Challenges in Achieving Collaboration in Clinical Practice: The Case of Norwegian Health Care

**DOI:** 10.5334/ijic.2217

**Published:** 2016-07-18

**Authors:** Sissel Steihaug, Anne-Kari Johannessen, Marian Ådnanes, Bård Paulsen, Russell Mannion

**Affiliations:** 1 Senior Researcher, MD, PhD, SINTEF Technology and Society, P.O. Box 124, Blindern, N-0314 Oslo, Norway; 2 Researcher, Nurse, PhD, Institutt HEL, HF, Høgskolen i Oslo og Akershus, Studiested Kjeller, Pb 4, St. Olavs plass, 0130 Oslo, Norway and Health Services Research Unit, Akershus University Hospital, Lørenskog, Norway; 3 Reseach Manager, PhD, SINTEF Technology and Society, P.O. Box 4760, N-7465 Trondheim, Norway; 4 Senior Researcher, Political Scientist, SINTEF Technology and Society, P.O. Box 4760, N-7465 Trondheim, Norway; 5 Professor of Health Systems, PhD, Health Services Management Centre, University of Birmingham, Park House, 40 Edgbaston Park Rd, Birmingham, B15 2RT, UK

**Keywords:** integrated care, inter-professional collaboration, patient provider relationships, clinical work, Norway

## Abstract

**Introduction:**

This article summarizes and synthesizes the findings of four separate but inter-linked empirical projects which explored challenges of collaboration in the Norwegian health system from the perspectives of providers and patients. The results of the four projects are summarised in eight articles.

**Methods:**

The eight articles constituted our empirical material. Meta-ethnography was used as a method to integrate, translate, and synthesize the themes and concepts contained in the articles in order to understand how challenges related to collaboration impact on clinical work.

**Results:**

Providers’ collaboration across all contexts was hampered by organizational and individual factors, including, differences in professional power, knowledge bases, and professional culture. The lack of appropriate collaboration between providers impeded clinical work. Mental health service users experienced fragmented services leading to insecurity and frustration. The lack of collaboration resulted in inadequate rehabilitation services and lengthened the institutional stay for older patients.

**Conclusion:**

Focusing on the different perspectives and the inequality in power between patients and healthcare providers and between different providers might contribute to a better environment for achieving appropriate collaboration. Organizational systems need to be redesigned to better nurture collaborative relationships and information sharing and support integrated working between providers, health care professionals and patients.

## Introduction

The health care system’s duty is to treat patients; i.e. clinical medical activity provided by various groups of healthcare professionals. In many countries, increasing numbers of older people and individuals living with chronic conditions in the community have created a need for better coordination and integration between different parts of the health system. In four separate but inter-linked empirical projects we explored the professional and organizational challenges to achieving collaboration in different health care settings in the Norwegian health system from the perspective of both providers and patients. In this article we explore the significance of these challenges for clinical work through a synthesis of the findings from across all four projects.

### Clinical work

The basic activity of healthcare services is clinical work with individual patients and their next-of-kin. Clinical practice includes discretional assessment and interpretation. A clinical conclusion is reached through logical reasoning based on, among other elements, clinical knowledge, i.e. the accumulated individual experience of work with patients. Communication is essential to delivering good quality medical care and a meaningful relationship between patient and provider is essential. Patients are all unique and individual patient needs are different. Providers need to ascertain an individual’s attitudes, values, and thoughts and give him or her the feeling of being understood. In addition to collaboration between patients and doctors, collaboration between providers is also required, especially when caring for older patients with complex problems and patients with chronic conditions, for instance people with mental disorders and/or substance abusers.

### Collaboration

Collaboration between providers can imply that providers from different specialities, disciplines or sectors work together. This includes a wide spectrum of activities – from simple electronically-conveyed messages or face-to-face encounters to comprehensive inter-professional, perhaps integrated, work. Integration implies a certain degree of collaboration among the parties who work together [1,2,3]. On a practical level it requires an effort to integrate and translate themes and schemes shared by different professional groups and the shared ownership of common goals, decision-making processes, and the integration of specialised professional knowledge and expertise. Barriers to overcome with regard to the successful integration between health care professionals include a blurring or misunderstanding of professional identities, roles and responsibilities [3,4,5]. A good knowledge of each other’s work, a culture of mutual respect and recognition of each other’s areas of expertise and competence, and the free and open exchange of information are also key elements. The significance of good relationships between providers and having sufficient time and resources for ongoing relationship-building has been well rehearsed in the literature [3]. Several empirical studies have also highlighted the key significance of effective organisational leadership, and appropriate funding arrangements for the achievement of successful collaboration and integration [3,6,7]. Singer et al. [8] recently addressed the concept of patient-centeredness in the provision of integrated care. In line with the clinical approach in the present study we also adopted a patient-centred perspective and included a focus on collaboration between patients and providers.

### The Norwegian healthcare system

Health care in Norway is divided into two broad delivery systems: the specialist and the primary healthcare system, each of which is subject to different funding systems, laws, and central regulations. Four regional health enterprises owned by the state are responsible for the provision of hospital services. Hospitals are financed by a combination of block grants and activity based financing, with hospital employees paid on the basis of a fixed salary. The 428 municipalities, which comprise the lowest governmental level, have responsibility for providing primary health care, long-term care services, home based care, and social care provision [9]. Health and social services are based on the classic Scandinavian Welfare model with financing and provision of universally accessible services to everyone. Nursing homes, home-based services and social care are public financed and mainly public provided but with an increasing part of private actors. As part of the Coordination Reform implemented in 2012 [10] specific responsibilities and resources are progressively being devolved from central to municipal government with the key aim of reducing hospital beds and widening access for health services within the municipality. Policies and programmes to achieve this aim are among other initiatives which established intermediate units between the two governmental levels, and the introduction of a daily penalty fee when municipalities are not able to receive those patients that are ready for discharge from hospital. Since 2001 all Norwegian citizens have been registered with a general practitioner (GP). GPs are in part paid by a capitation component depending on the number of patients on the list, and partly on the basis of fee-for-service.

As in other Western countries there have been a number of significant health care reforms in Norway over recent decades. The public sector modernisation, recommended by the Norwegian government, includes reorganisation inspired by core ideals in the New Public Management to achieve more cost-effective solutions in care provision [11,12]. These reforms involve among other factors a greater emphasis on measuring outcomes with the introduction of explicit standards, a range of performance metrics used to assess provider performance, and a greater degree of competition via the creation of quasi-market mechanisms. An example of this is the purchaser-provider split model which has a clear administrative distinction between those who assess the need for services and those who determine the scope of the services and provide the services. The contract contains detailed specifications from the purchaser and outcome control requires detailed reporting by the provider [11,12]. The increasing professional differentiation and sub-division of healthcare services in Norway has been allied with attempts to improve collaboration and coordination between health care services. Since 2001 patients requiring coordinated services have had a statutory right to an individual care plan (IP). The latest initiative for improving the coordination of services is the Coordination Reform [10].

There is a vast literature on the range of challenges in organisational and professional collaboration, but there has however, been a paucity of empirical research on how these problems impact on clinical work. In this article we draw on a range of empirical work undertaken in four different contexts in the Norwegian health care system which explored patient and provider experience of collaboration in real world contexts.

## Aim

The overall aim of this article is to explore how challenges of collaboration impact on clinical work through an examination of provider and patient experience and perceptions.

## Background and context

Below we summarise the key findings from an empirical research project – “The challenges of collaboration in an integrated health care system” - which was undertaken between 2010 and 2012. The study comprised four distinct but inter-linked sub-projects. The key elements of the main project are presented in Box 1.

BOX 1 **“The challenges of collaboration in an integrated health care system”**

The main study was designed as a multi-site-research project and explored collaboration in four specific healthcare contexts:

- an intermediate unit for older people
- mental health care services
- home-based rehabilitation services
- collaboration of GPs in the municipal care

Qualitative data were gathered through semi-structured interviews and participant observation as outlined in the Methods section (see Table 1). The findings from the study are summarised in eight articles, outlined below:

**Sub-project I** explored an intermediate unit recently established to improve the clinical pathway from hospital to home for patients aged 60 and over. The nurse-led unit had 15 beds, was located near a university hospital, and constituted collaboration between the hospital and four municipalities. The results of this study are summarised in three articles:

*Johannessen: Article I* explored the activities carried out and the conditions required to enable satisfactory work in the unit. The findings indicate that unfavourable environmental and adverse organisational factors exerted pressure on effective working and impeded patient clinical pathways [13].

*Johannessen: Article II* examined the unit’s role in a clinical pathway. Healthcare providers in the hospital, the intermediate unit and the municipalities had different opinions about who is a “suitable” patient for the unit as well as the most appropriate time for hospital discharge. This resulted in lengthy negotiations between the hospital and the unit [14].

*Johannessen: Article III* explored the significance of professional roles in collaboration on patient transitions from hospital to home via the intermediate care unit. Collaboration within the unit and between the healthcare institutions was primarily viewed as “a nursing matter”. Apart for the physician, all the healthcare providers perceived the level of collaboration in the unit as being ‘uni-disciplinary’ rather than ‘inter-professional’ [15].

**Sub-project II** explored young adult mental health service users’ care pathways and focused on factors associated with continuity and disruption of care. The municipalities have the responsibility for primary care, including primary mental care services. The specialised mental health service is integrated with and run according to the same principals as other specialised health care services. The results are summarised in two articles:

*Ådnanes: Article I.* Key obstacles to continuity of care included the mental health system’s lack of access to treatment, poor integration between different specialist services, and inadequate tools for coordination [16].

*Ådnanes: Article II.* Users’ perceptions of services were influenced by fragmented care and a lack of user involvement. Concurrent problems were viewed in isolation rather than treated holistically. Some patients felt rejected when seeking to participate in key decisions regarding their medicine, diagnoses and treatment. Developing good relationships between providers and patients was considered crucial to effective working but in practice proved difficult to achieve [17].

**Sub-project III** explored how rehabilitation work was perceived and delivered by front-line services in two boroughs in Oslo. Norwegian municipalities are required to offer social, psychosocial, or medical rehabilitation to all inhabitants requiring such services and to establish a coordinating unit for rehabilitation. The results are summarised in two articles:

*Steihaug: Article I.* Home-based rehabilitation received little attention in the boroughs, but participation in the project provided a broad discussion of rehabilitation. Starting with agreed policy guidelines and staff experience the researchers and borough staff jointly developed a model for organisation of and cooperation on rehabilitation [18].

*Steihaug: Article II.* Results show that patients were rarely rehabilitated at home. The purchaser-provider organisation of home-based services, the rushed nature of service delivery, and limited resources were reported to impede effective rehabilitation work. There was a discrepancy between the high level of ambition of the health authorities and how these could be achieved by practitioners on the ground [19].

**Subproject IV** explored the various contextual barriers that served to attenuate effective collaboration between GPs and other health professionals working in managed primary healthcare services. The results are summarised in one article:

*Paulsen*: Playing a key role, GPs share their treatment of patients with many collaborative partners. GP’s collaborative patterns are dependent on individual priorities according to personal interests, considerations of importance, personal affinities, time schedules and practical barriers related to differences concerning branch-related organization and funding. The different branches of primary health care are organized according to an internal rationale. Conflicting principles of organization and funding between branches served to block effective collaboration. Cross-level professional axes between professionally interrelated branches of primary and specialist care complicated collaborative relations within primary care itself [20].

## Material and methods

### Material

The eight articles arising from the project and outlined in Box 1 constitute the empirical material underpinning this article.

### Methods

We used the meta-ethnography method as originally developed by Noblit and Hare [21,22,23] as a guiding framework for integrating and synthesizing the findings across the eight articles. This method was developed for synthesizing published literature and involves taking relevant empirical studies to be synthesized, reading them repeatedly and noting down key concepts. The synthesis of these key concepts is achieved via a translation. The method comprises seven sequential phases as outlined in Box 2.

BOX 2 **Noblit and Hare’s 7 phases for conducting a meta-ethnography**

**Phase 1: Getting started** – identifying an intellectual interest that qualitative research might inform. This may be modified as interpretive accounts are read.

**Phase 2: Deciding what is relevant to initial interest** – defining the focus of the synthesis, locating relevant studies, and making decisions on inclusion and quality assessment.

**Phase 3: Reading the studies** – becoming familiar with the content and details in the included studies and beginning the process of extracting emerging themes.

**Phase 4: Determining how the studies are related** – creating a list of themes from all the articles, juxtaposing them and determining how they are related. This leads to initial assumptions about relations between studies.

**Phase 5: Translating the studies into one another** – the themes in each article and their interactions are compared and contrasted with the themes and their interaction in other articles. When studies are about similar issues, they can be synthesised as reciprocal translations. These direct translations may reveal that different concepts from one study are better than those in others in representing both studies. The translation makes it possible to establish new concepts and disentangle new relationships between concepts. These translations constitute the initial level of meta-ethnographic synthesis (first order analysis).

**Phase 6: Synthesizing translations** – this involves a continuous comparative analysis of texts until a comprehensive understanding of the phenomena is achieved. The synthesizing translation process is described as similar to general processes of qualitative research. Various translations can be compared with each other analyzing types of competing interpretation and translating them into each other, to produce a new interpretation (second order analysis).

**Phase 7: Expressing the synthesis** – for the proposed synthesis to be communicated effectively it needs to be expressed in a medium that takes account of the intended audience’s own culture and so uses concepts and language they can understand.

In practice these seven phases may occur in parallel or be overlapping.

### Accomplishment

Multidisciplinary teams are a useful approach to undertaking a meta-ethnography [22,23]. In the present study, the analysis was conducted by four researchers (SS, A-KJ, MÅ, BP) each with different specialist professional backgrounds, namely: Medicine, nursing, psychology, and political science. In addition, four researchers with different disciplinary backgrounds, including health services research, economics, sociology and political science were involved in the wider research group. The whole group met at half-yearly workshops throughout the period of research and openly debated and discussed the analysis, emerging findings and their interpretation.

#### Phase 1 – Getting started

A formal systematic searching of the literature was not required as we were synthesizing results from our own eight empirical articles. In several work-shops in the wider research group the authors discussed the content and findings of the eight articles in detail and agreed on their overall research quality based on, in addition to relevance, the following general criteria [22]:

- Are the objectives of the research clearly stated?
- Is the research design clearly specified and appropriate for the objectives?
- Do the researchers provide a clear account of the process by which their findings were produced?
- Do the researchers display enough data to support their interpretations and conclusions?
- Is the method of analysis appropriate and adequately explicated?

#### Phase 2 – Deciding what is relevant to initial interest

The participants of the wider research group jointly defined the focus of the study and developed the research question.

#### Phase 3 – Reading the articles

Even though the authors knew all the sub-projects fairly well we read all the eight articles thoroughly at the start of the process of analysis. We first extracted information on the context, methods, and informants in all the projects (see Table 1).

**Table 1:** Articles, informants, context, and methods in the four sub-projects.

Sub-project	Article	Informants Providers/patients	Context	Recruiting informants	Data collection

1	Johannessen I	16/8	Intermediate unit	Strategic sample	Individual interviews Group interviews Observations in collaboration meetings
	Johannessen II	38/8	Intermediate unit Hospital Four municipalities	Strategic sample Snow ball sampling	Individual interviews Observations in collaboration meetings
	Johannessen III	38/0	Intermediate unit Hospital Four municipalities	Strategic sample Snow ball sampling	Individual interviews Observations in collaboration meetings
2	Ådnanes I Ådnanes II	0/9	Mental health field	Recruited from municipal services, user organization, secondary school, and snow ball sampling	Repeated individual interviews
3	Steihaug I Steihaug II	24/0	Home-based services	Strategic sample	Individual interviews Group interviews
4	Paulsen	10/0	Primary health care	Strategic sample	Individual interviews

This step also included beginning the process of extracting themes in the articles. Each researcher extracted her/his own emerging themes using as far as possible the terms used in the original papers. We extracted eleven themes related to the challenges to collaboration in clinical work from across the eight articles.

#### Phase 4 – Determining how the studies are related

Preparation for comparison between studies requires listening and juxtaposing the themes and concepts used in each account. For comparing the themes and concepts in one article with themes and concepts in others we chose to use grids with the articles located along the X-axis and the themes located along the Y-axis. We compared studies, and our initial broad grouping of themes was gradually refined by merging, deleting, and establishing categories. Through group negotiation and discussion we eventually agreed on the three key themes: “collaboration with patients and between providers”; “different professional view”; “the significance of organisation for collaboration”.

#### Phase 5 – Translating the studies into each another

We explored each of the three key themes identified across all of the articles, see our final grid; Table 2. We chose an index study [14], characterised by high methodological quality, a broad data base, and systematic presentation, as a starting point. For each study we examined in detail the issues related to the given concept, for example “different professional view”. We related the core content issue of each paper to each other horizontally. The interpretations in the right hand column in Table 2 are results of our translating the studies into each other (the first order analyses).

**Table 2:** Translating the studies into each another – first order analysis.

Johannessen I	Johannessen II	Johannessen III	Ådnanes I	Ådnanes II	Steihaug I	Steihaug II	Paulsen	Our Translation

The nurses in the intermediate unit collaborated appropriately and the patient felt well cared for.	The collaboration between different organisational levels was mainly a “nursing thing”.	Nurses had an inclusive collaborative culture which excluded other professional groups. Inter-professional collaboration was poor.	Repeated disruptions occured in the care pathway when the patient was transferred from one service unit or level to another. Individual plans were established, but did not worl as intended.	A lack of appropriate cooperation was found between provider and patient and between providers.	In the relevant rehabilitation case the different services were established, but the providers did not collaborate appropriately. The patient was not involved in developing her rehabilitation plan.	Home-based rehabilitation was afforded little attention and seldom occured in practice. The providers needed better inter-professional collaboration but framework condition were locking.	Several providers wished to collaborate with GPs, but the GPs had to make priorities. GPs preffered to collabrates with their professional hospital colleagues.	*Good professionals collaboration was achieved, but the providers did not succeed achieving effective inter-professional collaboration. Collaboration between patients and providers in mental health was often poor.*
A nursing perspective and nursing activities dominated in the unit. Physio-therapistis and occupational therapists missed more rehabilitation.	There was disagreement between the collaborative partners about inclusion criteria and “suitable” patients for the unit and about what were the unit’s role and tasks.	Different professionals had different opinions about inter-professional collaboration. The physician performed medical work, while the others wanted her to contribute to rehabilitation. There was medical dominance in inter-professional meetings.	Different views of “treatment” complicated the collaboration between the primary health care and the specialist services.	Patients and providers had different understanding of illnesses, diagnoses, treatment, and patient involvment. Providers in specialist services were regarded as “therapists”, while providers in primary services were “helpers”.	Different professional groups disagreed as to what rehabilitation is and had different foci in the rehabilitation process. Providers and managers disagreed on the aim of reducing the face-to-face communication in the home-based services.	Different occupational groups worked seperately with different methods againts different goals in the relevant rehabilitation case. Providers in different positions and different levels disagreed about how best to prioritise rehabilitation.	Different professionals in the municipality had different views on inter-professional collaboration and different views on the GP’s role as a collaborative partner.	*Different professionals and different units had a range of conflicting perspectives and worked towards different goals.*
The intermediate unit consituted collaboration between a hospital and four municipalities. This gives rise to a new collaboration interface in patients discharge.	The three organisational levels’ differnet aims and tasks hampered the collaboration. The hospital aimes to discharge patients at once they were ready, the unit wanted patients needing rehabilitation, and the minicipality wanted the patients to stay as long as possible.		Different symptoms were treated in different department in hospitals or in different hospitals. There were lacking collaboration structures between different department in specialist services and between the two levels.	Specialist services were responsible for “treatment” and the municipality for “following up” patients. The users found that their services were not coordinated appropriately between units in specialist services or between levels, Coordinating tools were found inadequate.	A coordinating units for rehabilitation was lacking in the boroughs.	Purchaser/provider organisation’s splitting up work into smaller, measurable units hampered inter-professional collaboration in rehabilitation work.	Each professional group had their own organisation without appropriate coordinating structure in place in the municipalities. Purchaser provider splits organisation hampered collaboration.	*Organisation principles served as a barrier to appropriate collaboration between departments and between professionals.*

#### Phase 6 – Synthesizing translation

For the synthesizing translation - the second order analysis - we used a method for systematic text condensation (STC). This comprises a four-step, cross-case method for thematic analysis suitable for developing descriptions of experiences within a field, in this case, how challenges of collaboration impact on clinical practice [24]. In the first step we re-read all the articles with the three concepts developed in the first order analyses as a starting point (see the right hand column in Table 2). The second analytic stage included identifying meaning units related to each of these three topics. A meaning unit is a text element relevant for the problem to be addressed, for instance “Mental health service users spoke about the importance of being “seen” and cared about, feeling respected and taken seriously”. We analyzed each of the three concepts separately. Meaning units were developed, refined, and systematized to codes, for example “insufficient collaboration between patent and provider”. These codes were assembled into code groups under appropriate headings, for example “neither inter-professional collaboration nor collaboration with patients”. In the third stage we analyzed and condensed the contents of each code group, and the fourth stage was to summaries the condensed text in all the groups into a précis, an analytical text that constitutes our results. An appropriate heading was developed, for instance: “A lack of collaboration”. Quotations in the texts were selected to both illustrate and illuminate the key issues that were surfaced.

#### Phase 7 – expressing the synthesis

The results of our first order analyses - translating the studies into each another – are presented in Table 2. Results of the second order analyses are presented under the heading “Results” below.

The third row, third column in the table is empty because the article “Johannessen III” does not address organizational issues.

## Results

### A lack of collaboration

A recurring theme across the four contexts is the lack of appropriate collaboration: between patients and providers [16,17] and between providers [13,14,15,16,17,18,19,20]. The patients wanted better continuity and predictability in their services [17]. The employees described the need for inter-professional collaboration, but they did not succeed in achieving this [14,15,18,19,20]. In both the rehabilitation work and in mental health the providers failed to develop appropriate individual care plans (IP) [16,18]. The consequences seemed to be worst for young mental health service users. They reported examples of fragmented health services and situations in which they had to deal with multiple practitioners not collaborating well [16,17]. The patients with additional substance abuse problems, experienced extreme discontinuity of care because drug problems and mental health issues were treated by different parts of the specialist health services [16,17]. One participant’s statement clearly illustrated this point [17]:

“I miss a combined treatment. Drug abuse is at most only a symptom of something else. They have not realized that probably 99 percent of those with drug problems are also suffering from psychological problems. So, if you attend substance abuse treatment, you take away the symptom, but all the reasons why people take drugs, are still there. No places are suitable, for either they relate to the psychological problems or they relate to the substance abuse problems. It is frustrating.”

The mental health users reported both good and poor relationships with the provider [17]. They emphasised the importance of being seen, understood, and taken seriously and told powerful narratives about situations when this was not the case. Good patient-provider relationships required, according to the patients “good chemistry,” trust, and continuity.

Many mental health service users missed information and more influence on their services [17]. Several reported about how they “were admitted” and “were discharged” without being consulted.

The intermediate unit with different professionals working together to get patients “back on their feet” seemed to be a perfect place for inter-professional collaboration, but they were not successful in achieving this [15]. The employees reported that better collaboration could have prevented time-consuming discussions and that the patients’ care plans could have been more adjusted to the patients’ needs. The nurses described an inclusive and educational nursing collaboration in the unit, while the physiotherapists and the occupational therapists desired better inter-professional working. Providers in the municipal services also reported examples of poor collaboration [18,19,20]. The physiotherapists and the occupational therapists in the two boroughs reported limited inter-professional collaboration with their nursing colleagues and argued that the rehabilitation work suffered from lacking collaboration [15,18]. Despite the fact that municipalities are required by law to provide rehabilitation, patients were rarely rehabilitated in their own home [18]. One rehabilitation case was found in one borough during one year, but the different professionals involved did not collaborate. Work with an IP was not initiated until several months had elapsed and the patient was not involved in decision made over her care needs. The providers emphasized that nobody had the overall responsibility for coordinating the work with an IP.

Different providers in the municipality reported wanting to collaborate with local GPs [18,20], but GPs interviewed said that they were too busy to meet these demands and had to make priorities. Several professionals in the municipalities reported that they are more in touch with their professional colleagues in specialist services than with other professional groups on the same level [13,14,18,20].

### A case looks different from different perspectives

The differing perspectives on illness and treatment seemed to be one reason for a the existence of poor relationships between mental health service users and their providers. While professionals emphasised the need for providing an accurate diagnosis for the patient, the patients themselves were far more concerned about the cause of their mental health problems rather than being told the a name of their problems. Most of patients had received a diagnosis, but individual patients reacted differently to their diagnosis. One patient diagnosed with a personality disorder, for example, disagreed with the providers’ diagnosis and did not find the diagnosis helpful at all. In fact she experienced not being taken seriously because her expressed opinion, for instance criticisms of the health care system or scepticism towards medication treatment, was interpreted as symptomatic of her personality disorder. She did not feel her needs were being “met”. Most of patients were medicated and some had felt pressured to take medicines. Several were sceptical about the use and impact of the medicines they were prescribed.

A recurring theme across the articles was the finding that different professional groups had different perspectives on illness and treatment and how they gave priority to different patients’ needs. The significance of profession and occupational position was clearly demonstrated across different contexts. Both in the intermediate unit [15] and the municipalities [18,19,20] physicians, nurses and physiotherapists revealed different understandings of their own and others’ roles and tasks. Rehabilitation is clearly defined, but nurses, physiotherapists, and occupational therapists disagreed about what rehabilitation is and ought to be [13,18,19]. In the intermediate unit the physician’s colleagues reported that she used to set the agenda in the inter-professional meetings and that medical issues often dominated at the expense of discussions concerning rehabilitation and recovery [15]. Health professionals at different levels in the hierarchy and in different positions also held different perspectives. In spite of the fact that the intermediate units’ collaboration with the hospital and the municipalities consisted primarily of collaboration between nurses, the providers had different opinions about what kinds of tasks the unit should perform [14]. The hospital and municipal informants maintained that the unit’s main task was rehabilitation while on the other hand employees in the unit reported that their role also included medical treatment, and that patient treatment should not be completed before discharge to the unit. Several providers reported that such disagreements often complicated and lengthened time of the patients’ discharge pathway. This is clearly illustrated by a hospital nurse who reported:

“We had a very suitable patient for the unit, but she was regarded as having completed treatment and so there was no place for her. There are written rules about how to define a patient who is ready to be discharged, but they don’t necessarily work in practice”.

In the municipalities, nurses on the managerial level placed less emphasis on home based rehabilitation than nurses on the clinical level [18,19]. The providers argued that this could imply that patients needing rehabilitation were not identified.

### Organise according to the providers’ rather than the patient’s needs’

Significance of organisation conditions for clinical work and collaboration was evident in all of the sub-projects. Most clearly this was the case in the specialist mental health services [16]. The treatment pathways for young people with severe mental illness, especially when combined with substance abuse problems, were characterized by frequent transfers between services because their individual problems were treated separately by different units in specialist health services. Several reported that they were transferred from one unit to another without collaboration between providers in the different units. The patients also described poor coordination between specialist services and primary care. Several reported for instance that primary care providers and hospital providers could disagree about patients’ need for hospital admittance or discharged. Most of patients had an IP, but they still experienced discontinuity in service delivery. Many described the negative effects of this uncertainty and unpredictability. One patient reported:

“You never know what happens next; I do not know what happens this summer, I do not know what happens next fall. Nothing! I only know that the providers I now have will quit their job. I do not know if I should continue treatment in department x or not.”

GPs and their collaborative partners in the municipalities, primary the home-based services, highlighted that the home-based services’ purchaser provider split organization was a hindrance to effective collaboration in practical work [18,19,20]. Historically, GPs could phone the home-based services and ask them to take blood-test. Now they had to make an application to the purchaser office [20]. “We cannot always wait for that”, a GP related. A number of providers told that the purchaser provider split model hampered the rehabilitation work because they missed being able to make their own assessments and to give priority to tasks other than those on the contract. As one physiotherapist complained [19]:

“It’s a problem that home nurses work according to decisions taken by others than the providers of the services, and they aren’t allowed to do anything other than what is stated in the decision. Needs change constantly during a rehabilitation process, and decisions have to be constantly altered – on application – by the service providers. We have to deal with a lot of red tape in order to get anything done. Collaboration has got so cumbersome since the purchaser-provider organisation was brought in.”

It was also reported out that no single authority in the municipality had the overall responsibility for promoting and ensuring effective collaboration.

## Discussion

The overall aim of this article is to explore how challenges of collaboration impact on clinical work in healthcare services in Norway across four different healthcare contexts: (i) intermediate unit for older people, (ii) mental health care services (iii) home-based rehabilitation, and (iv) GPs’ collaboration in municipal care. The results from the meta-analysis of the eight articles highlight a lack of effective collaboration between patients and providers and between health professionals and expose a range of barriers to collaboration. Providers described how poor collaboration made good clinical work difficult, and patients reported challenging meetings with providers and experienced a lack of holistic care. Both organizational and individual level factors were attributed to impede collaboration. Below, we discuss the challenges in collaboration arising from our analysis and detail how they impeded effective clinical work.

### Insufficient collaboration hampers clinical work

#### Collaboration between patients and providers – the essence of health care

Relationships and collaboration between patient and provider are central to good quality clinical practice. The mental health service users articulated narratives relating to both good and poor collaboration with providers and highlighted how an unsatisfactory relationship sometimes resulted in them feeling rejected, insecure, and their needs not being adequately met. Patients and providers sometimes disagreed on the significance of the diagnosis and this disagreement complicated the collaboration. One patient reported that she felt rejected when her complaints were understood in light of her diagnosis. This aligns with the findings of a recent research project on user involvement in health services [25]. Mental health service users experienced that their complaint about the healthcare system were often attributed to mental health problems. Psychologists and psychiatrists aim to make diagnoses. Several patients in our study did not feel helped by a diagnosis, for instance “depression”; they wanted an explanation of the symptoms and an understanding of the reasons why the symptoms had developed; they needed to understand themselves. These different preferences may be attributed to different professional knowledge bases. As a medical specialty, the field of psychiatry is based on a medical logic in which mental disorders are classified into stable, universal categories - diagnoses - where one diagnosis is associated with specific treatment methods with predicted effects [26,27]. In clinical medicine, the point is not, however, to understand the illness but the ill person in a more holistic sense - to understand the person as a social and intentional human being that cannot be understood in isolation from his or her social environment. The patients’ ambivalence regarding use of medicine does perhaps reflect that the biomedical model of disease does not always fit with their experience of their illness.

#### Collaboration between professionals in a fragmented health care system

Providers in mental health services were not interviewed. Patients, however, reported issues related to fragmented services and a lack of collaboration between providers within the specialist mental health services and between specialist services and primary care. Ramsdal describes a schism between specialist and primary mental health services in Norway because the two have developed separately from one other based on different knowledge bases and different organization and management principles [28]. This description concurs with the perspective of that of Roger and Pilgrim [27]. In our study providers’ differing views on patients’ need for hospital admittance may be an example and this is presumed to complicate collaboration and prevent more integrated services. A small number of patients reported that services forming a whole despite serious mental problems and many providers involved. This indicates that some cases of successfully integrated services occurred.

The professional groups in the intermediate unit were not successful in implementing appropriate inter-professional collaboration despite a favourable context. Different foci, cultures and ideologies across the different professional groups appeared to impede their daily work. The nurses emphasized the caring aspect, while the physiotherapist and occupational therapist tended to focus on the need for rehabilitation, and the physician was more focused on medical treatment. Insufficient inter-disciplinary collaboration seemed to hamper work with patients. In the municipality the nurses, physiotherapist and occupational therapist disagreed about what rehabilitation entailed, despite the fact that a national definition exists. This may be why home-based rehabilitation was rarely provided and only one patient who was rehabilitated at home was identified in the study. Aligning with other studies [3,4,5,29,30], these results indicate that different bases of professional knowledge and different understanding of professional demarcation roles and tasks were important barriers to effective collaboration. With a long patient list and a diversified patient perspective, GPs may consider it inappropriate to give priority to one particular patient group – irrespective of requests from other groups of health professionals. The nurses in primary care wanted more collaboration but GSs mentioned time pressure as one obstacle. Results of a Swedish study indicate that nurses are slightly more positive about collaboration than GP’s and that a positive attitude to collaboration is part of nurses’ professional role to a larger extent than the GPs’ [31]. This may have influenced the GPs’ modest wish for inter-professional collaboration and preference to collaborate with providers in specialist services [32]

Disagreement also occurred within one professional group taking on different roles. In accordance with the findings of another Norwegian study [5] it appears that our informants’ perspectives were dependent on their position. Despite the fact that managers from the hospital and the municipalities had agreed on inclusion criteria for the intermediate unit, the nurses at different levels (hospital, unit, and municipality) had different opinions of “suitable” patients and what rehabilitation entailed. This could, according to the providers, postpone admittance to and discharge from the unit.

Differences in positional and economic power may also lead to significant barriers to inter-professional collaboration. The physician’s influence in the intermediate unit may well have a connection with the authority and power physicians have traditionally enjoyed through their use of scientific and diagnostic language, their monopoly on to defining what constitutes disease and illness and what constitutes knowledge and expertise in clinical practice. [33,29]. More listening to the other professionals’ perspective might have made the physician a better collaborating partner, for instance in rehabilitation work. In collaboration between GPs and specialists in mental health, GPs seem to feel inferior; they want to be regarded as competent colleagues by the specialists and to be accorded the same level of respect that specialists show each other [32].

Inter-professional collaboration on patients is personally and professionally demanding, takes time, and presupposes that health care professions both know and trust each other. [3]. Developing relationships and trust between providers need time and knowing each other. This is impeded by time pressures and lack of places to meet on a face to face basis.

### Organisation provides a framework for collaboration

Collaboration between professionals was hampered by organizational restraints, both horizontally and vertically and the informants’ narratives illustrated how this impeded clinical work. Patients in mental health specialist services reported systematic discontinuity with ever new providers. For instance were different symptoms treated in different departments in specialist services without collaboration between providers in the different departments. Ramsdal [28] attributed this organisation to the development of knowledge, skills, and professional composition within a particular field over time. This can be seen as consequence of the rapid increase in knowledge leading to professional differentiation and the sub-division into several sub-specialities, for example departments for substance abuse treatment, treatment for eating disorders, personality disorders and so forth. The extreme specialisation bring along that more and more doctors can get to participate in a constantly lesser part of what for the patient should be unifying help.

Despite that the intermediate unit, the hospital and the municipalities had an agreement on co-operation they could not agree on the basis and the overall aim of their work. When the course of treatment was completed, it was presumed that the hospital had fulfilled its tasks. The hospital must have free beds and the patient should be discharged as soon as possible. This was not necessarily a “suitable” patient for the unit. They wanted patients with potential for improving their health during the stay in the unit and patients needing inter-professional collaboration. The municipality was committed to receiving patients ready to be discharged. Christensen et al. [34] describe how different organizational goals can express conflicting interests and result in tensions between cooperative participants. The intermediate unit and its collaborative partner, the hospital and the four municipalities had different commitments, goals, and tasks, and the results illustrate how conflicting goals created tension and challenged the cooperation between providers on the different levels and probably extend the patients’ discharge pathway from hospital to home.

Also within primary health care we identified considerable barriers to effective collaboration across institutions. Primary health care in Norway is poorly integrated and is best characterized as a conglomerate of loosely coupled units organized according to professional groups, i.e., home nurses, home helpers, physio- and occupational therapy, GPs etc. Several emphasised the purchaser provider split as causing challenges in clinical work and collaboration on patients. Vabø [12] argue that steps taken to make home care services more transparent and reliable have made them less sensitive to the particular needs of individual service recipients. Clinical practice implies complicated and interwoven work tasks. Making a clinical assessment requires clinical competency and must be done by professionals who monitor the patient and the patient’s varying needs. In a purchaser provider organisation decisions concerning services are taken according to standardised time estimates per part-task by someone who is not close to the patient. It requires clinical work to be broken down into smaller, measurable units. The basis for the purchaser provider split organisation is justice, objectivity and impersonality. In clinical work, however, the key issue is for the provider to help the patient which requests that they together understand and agree on how this help ought to be performed. Similar organizational changes characterise health services in the in UK [30], Sweden [35], and Canada [3].

### Collaboration in clinical work – how and when?

The health care exists for the patients and the patient provider relationship is the core of clinical work. Fragmented healthcare services require effective collaboration. Strong support in both national reforms and legislation and various strategies and means have been used in Norway to improve collaboration in the healthcare system. Thus far, it has, however, had only a limited impact on for example the use of IP [35,36,37]. Holum [38] found a lack of time, muddled responsibility, and lack of rights and resources for the patient as reasons for the limited use of IP. Ahgren [35] argues that the modest effect of IP in practice is due to the fact that very little is done to change the existing fragmentation by means of organizing collaboration between care providers involved. Health systems integration requires policies and management practices that support relationship-building and information-sharing across organisational and professional boundaries [3]. Framing conditions and methods for developing collaboration is a responsibility for managers at different levels of the health system and presupposes managers’ thorough knowledge about and understanding of clinical work. The importance of including the clinical level in planning and implementing means for collaboration is emphasised because they are at the core of clinical work [35,10,18,29]. On the other hand, if national policies are lacking, the collaboration arrangements in healthcare could develop in different ways with accordingly various effect for patients involved.

Collaboration is often represented as a “must”. We argue that the main questions ought to be what should be done for giving the patients the best possible services within available resources. The degree of collaboration itself does not appear to predict clinical outcome [39]. Collaboration is time- and resource consuming and a balance between time-consuming and time-saving is needed [31].

### Methodological considerations

We used meta-ethnography as a method to synthesize the results across our eight articles focusing on how key barriers to collaboration can impact on clinical practice from the perspective of patients and providers. We found the method well suited to this task. However, the analysis process was time-consuming; we had several discussions in developing the three main themes in the first order analysis and even more discussions in the text condensation process in second order analysis. There were, however, few problems in agreeing on the results. This may be due to the fact that we all knew all the sub-projects in advance though repeated discussions in the research group. A risk with knowing the projects so well could be that the researchers held common biases or took things for granted. The four participants in the half yearly workshops, however, asked questions, raised objections, and brought new perspectives. A strength of the study is that four different contexts were studied and a wide spectrum of professionals interviewed but a study limitation was that there were relatively few participants in some of the studies. In the mental health study a strength was that the patients were interviewed four times, a limitation was that providers were not included. Only the study of the intermediate unit was based on both observations and interviews with patients and providers. Observation as a method in the other studies might have brought valuable additional information.

The four researchers coming from different professions and perspectives performed the data analyses according to the meta-ethnography methods. Phase 5 in the meta-ethnography method – translating the studies into each other – revealed that numerous phenomena were found in all contexts and that new concepts and new relationships between the concepts could be established. In phase 6 – syntesising translation – we analysed the text of all the articles using the STC method. This allowed us to interpret concepts that encompassed more than one of the studies being synthesized. The significance for collaboration of power inequalities between different professionals was for instance clearly expressed in only one of the original projects [15] but through the synthesizing translation we found that power inequality occurred in all of the contexts: between providers in specialist and primary mental health care, between purchasers and providers in rehabilitation work, and between GPs and both their collaboration partners in the municipality and their colleagues in special health services.

The study’s new and original contribution to knowledge in this field is that our findings reveal how challenges to collaboration are intimately connected to a range of organizational factors across the four contexts under study. Patients’ and care providers’ experiences yielded bottom up perspective on how organizational conditions influence clinical work. Despite the study’s limitations we argue that our results have important lessons for other contexts and countries grappling with the design and operation of integrated care services in publicly funded and provided systems.

## Conclusions

A range of both organizational and individual factors appear key to achieving collaboration in clinical practice. We have argued that challenges in collaboration are also due to differences, disagreement and conflicts, which are often implicit or even unconscious. Providers’ conflicting perspectives may indicate, as noted in the wider literature [3,4,5], problems with viewing issues from another party’s perspective as healthcare professionals are trained in one kind of logic and reasoning. If the collaborating parties can manage to surface differences and disagreements we argue that collaboration will be more easily accomplished. Focusing on inequalities and asymmetries in power and influence between the patient and the healthcare provider and between different providers might contribute towards the design of a better framework for communication and collaboration between the different actors. Organizational structures in the health system need to be redesigned so as to better nurture collaborative relationships which support integrated working and decision-making between providers, health care professionals and patients.
